# Demography, trade and state power: a tripartite model of medieval farming/language dispersals in the Ryukyu Islands

**DOI:** 10.1017/ehs.2022.1

**Published:** 2022-01-26

**Authors:** Aleksandra Jarosz, Martine Robbeets, Ricardo Fernandes, Hiroto Takamiya, Akito Shinzato, Naoko Nakamura, Maria Shinoto, Mark Hudson

**Affiliations:** 1 Faculty of Humanities, Nicolaus Copernicus University, Toruń, Poland; 2 Archaeolinguistics Research Group, Department of Archaeology, Max Planck Institute for the Science of Human History, Jena, Germany; 3 Department of Archaeology, Max Planck Institute for the Science of Human History, Jena, Germany; 4 School of Archaeology, University of Oxford, Oxford, UK; 5 Faculty of Arts, Masaryk University, Brno, Czech Republic; 6 Research Center for the Pacific Islands, Kagoshima University, Kagoshima 890-8580, Japan; 7 Research Center for Buried Cultural Properties, Kumamoto University, Kumamoto, Japan; 8 Research Center for Archaeology, Kagoshima University, Kagoshima, Japan; 9 Institut für Ur- und Frühgeschichte, Zentrum für Altertumswissenschaften, Universität Heidelberg, Heidelberg, Germany; 10 Institut d'Asie Orientale, ENS de Lyon, Lyon, France

**Keywords:** Farming/language dispersals, trade, state space, Middle Ages, Ryukyu Islands

## Abstract

Hunter–gatherer occupations of small islands are rare in world prehistory and it is widely accepted that island settlement is facilitated by agriculture. The Ryukyu Islands contradict that understanding on two counts: not only did they have a long history of hunter–gatherer settlement, but they also have a very late date for the onset of agriculture, which only reached the archipelago between the eighth and thirteenth centuries AD. Here, we combine archaeology and linguistics to propose a tripartite model for the spread of agriculture and Ryukyuan languages to the Ryukyu Islands. Employing demographic growth, trade/piracy and the political influence of neighbouring states, this model provides a synthetic yet flexible understanding of farming/language dispersals in the Ryukyus within the complex historical background of medieval East Asia.

## Graphical abstract

**Figure:**
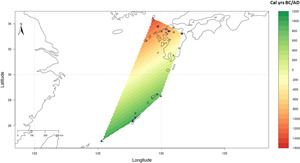

**Social media summary:** Agriculture and the Ryukyuan languages spread to the Ryukyu Islands in the medieval period through a complex historical process involving demographic growth, trade/piracy and the shifting power networks of neighbouring states.

## Introduction

**Figure 1.**
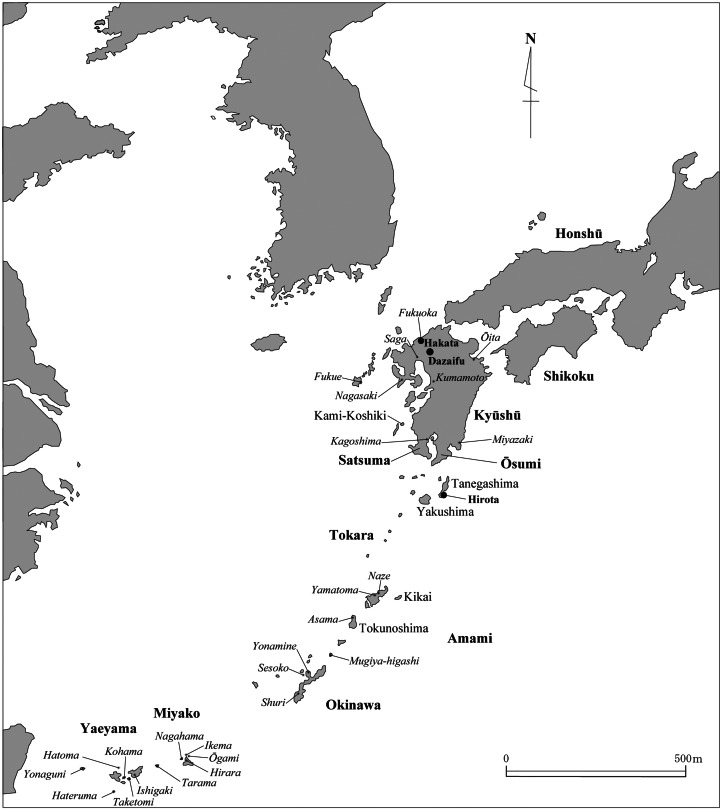
The Ryukyu Islands and Kyushu with main sites and topolects mentioned in the text. Topolects are shown in italics.

Small islands less than about 1700 km^2^ were rarely settled by humans prior to agriculture (Keegan & Diamond, 1987). Besides very large islands, a few exceptions comprise islands which are (a) close to large landmasses, (b) have abundant marine mammals and the technology to exploit them, (c) have translocated plants or animals or (d) display a combination of (a)–(c). From this perspective, the Ryukyu islands are highly unusual in world prehistory: although apparently lacking these exceptions – with the possible exception of the translocation of wild boar (Kawamura et al., 2016) – the Ryukyus were settled by hunter–gatherers in the Upper Palaeolithic (Kaifu et al., 2015; Fujita et al., 2016; Takamiya et al., 2016). Recent discoveries suggest probable continuous hunter–gatherer settlement of the Amami and Okinawa archipelagos since at least 7000 years ago (Takamiya, 2021; Takamiya et al., 2019). A similar antiquity may exist for the Yaeyama archipelago (Yamagiwa et al., 2019; Figure 1). However, the Ryukyus are *doubly* unusual because of the very late arrival of farming. Cereal farming of millet, rice, barley and wheat reached northern Kyushu around 1000 BC and southern Kyushu a few centuries later, but only spread to Amami in the eighth to twelfth, Okinawa in the tenth to twelfth, and the southern Ryukyus in the twelfth to thirteenth centuries AD. This was one of the latest primary farming dispersals in Eurasia, certainly in temperate or sub-tropical latitudes. If small island settlement is easier with agriculture, why was farming not adopted more quickly in the Ryukyus? Conversely, what historical conditions led to the eventual dispersal of agriculture there?

There is little theoretical research attempting to explain why agriculture and the Ryukyuan languages spread to the Ryukyus. Following the forced incorporation of the Ryukyu kingdom into Japan in 1879, early academic work reflected colonial discourse wherein Okinawan culture was to be established as an exotic subset of Japanese culture (Christy, 1993; Oguma, 2002). A typical example was the ‘Ocean Road’ hypothesis of folklorist Kunio Yanagita (1961), who proposed that rice had reached Japan *from* the Ryukyus. Despite its influence in Japanese studies, there is no archaeological evidence to support Yanagita's theory; it is clear that rice and other cereals spread south from Japan (Takamiya, 2001, 2021; Takamiya & Nakamura, 2021). Until archaeobotanical research was begun in the 1990s by one of the present authors (HT), comments about early farming in the Ryukyus were mainly based on speculation such as site locations and the possible function of stone tools and other artefacts. This type of approach remains common. Ishidō (2014) argues that the disappearance of grinding stones from Tanegashima Island during the Late Yayoi–Kofun periods suggests that nuts were no longer utilised and that rice was therefore being cultivated (see Table 1 for chronology). On neighbouring Yakushima, in contrast, the continued dominance of grinding stones suggests to Ishidō a continued hunter–gatherer occupation during the Kofun. In such writings, evidence for cultural interaction between the Ryukyus and Japan is often linked to farming on the assumption that hunter–gatherers would quickly adopt agriculture once available. In contradiction to Ishidō, Hashimoto (2012, p. 22) proposes that, since Yayoi pottery spread to Tanegashima by the Middle Yayoi, agriculture was probably accepted there by that time, but that the minor ceramic influences from Kyushu to Tanegashima during the Late Yayoi–Kofun eras suggest the islanders later quit farming and continued hunter–gathering.

**Table 1.** Overview of historical periodisation in the Ryukyus and Kyushu. Note that periodisation between the three regions does not always correspond exactly. While conventional historiography sets the division between Late Antiquity and the Middle Ages in mainland Japan at 1185, for convenience we use the term ‘medieval’ to refer to changes straddling this date in the Ryukyus

AD/BC	Central Ryukyus		Southern Ryukyus	Kyushu
ca. 11/12 – 15 centuries AD	Gusuku		Gusuku (Suku)	Kamakura to Muromachi
AD 600	Shellmidden Period	Late 2	Late Neolithic (non-ceramic)	Asuka to Heian
600 BC		Late 1		Yayoi to Kofun
1000 BC		Early 5		Final Jomon
2000 BC		Early 4	Early Neolithic (Shimotabaru)	Late Jomon
3000 BC		Early 3	?	Middle Jomon
4000 BC		Early 2		Early Jomon
5000 BC		Early 1		
		Beginning of pottery culture?		Initial Jomon
8000 BC	Palaeolithic		Palaeolithic	Incipient Jomon
30,000 BC				Palaeolithic

With poor soils and little available water, the Ryukyu Islands were not suited to irrigated cereal agriculture (Pearson, 2013, pp. 24–27). In order to understand why cereal agriculture eventually spread to the Ryukyus in the medieval period, there is a need to consider the linguistic and population histories of the islands.

**Figure 2.**
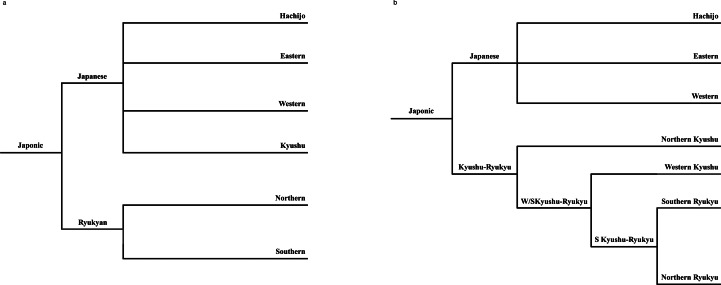
A comparison of the standard classification of Japonic (a) with the revised classification adapted from Igarashi (2018) (b).

Although the standard view is that the Ryukyuan languages form a first-order sister clade with Japanese–Kyushu in the Japonic tree (Katō, 1977; Lee & Hasegawa, 2011; Pellard, 2015; Robbeets et al., 2021), some scholars have challenged this view by suggesting that Ryukyuan is an offshoot of a first-order Kyushu–Ryukyu clade (Unger, 2009, p. 105; Igarashi 2017, pp. 5–6; de Boer, 2020, pp. 56–57). Figure 2 compares the standard classification (a) with the revised one (b). Since we know that Proto-Ryukyuan was originally situated on Kyushu and that Proto-Ryukyuan and Japanese remained in intensive contact until at least the eighth to ninth centuries AD (Pellard, 2015), it is not unlikely that the standard classification of the Kyushu dialects within the Japanese clade is a misinterpretation owing to strong interdialectal borrowing. Therefore, even if the high branch probabilities in a recent Bayesian approach (Robbeets et al., 2021) support the model proposed by Pellard (2015), Unger (2009), Igarashi (2017) and de Boer (2020, in press) may be right in suggesting that the Kyushu dialects should be grouped with Ryukyuan instead.

Most linguists (Hattori, 1976a, 1976b; Thorpe, 1983; Serafim, 2003; Bentley, 2008; Pellard, 2015; Igarashi, 2017, 2018; Jarosz, 2019, to name a few) accept that Ryukyuan originated on Kyushu, following which the actual migration of Ryukyuan speakers to the Ryukyus occurred. Because the Ryukyuan languages form a single branch, it has been inferred that ‘the settlement of the Ryukyus was not achieved by different waves of migration at different periods and from different places, but probably by one or a few moves from a single area, and within a rather narrow time frame’ (Pellard, 2015, p. 25). The shape of the Ryukyuan tree does not support a completely linear dispersal from north to south, but various proposals have been made as to the order and branching of settlement (Pellard, 2015, pp. 25–26)

While de Boer (in press) and Unger (2009, p. 100, 104–105) suggest a date of around the tenth century for the split between Ryukyuan and Kyushu Japanese, most researchers supporting the classical tree structure estimate that Ryukyuan split from Japanese before the seventh century: between AD 1 and 500 according to Hattori (1976a, p. 43), before AD 700 according to Serafim (2008, p. 98) and before AD 550 according to Frellesvig and Whitman (2008) and as concluded from Miyake (2003, p. 132–133). However, Hattori's dating is based on lexicostatistics, a controversial dating method, while the other linguists propose a *terminus ante quem*, a ceiling but not a floor for the time-depth of separation. Since we know that Proto-Ryukyuan and Japanese remained in relatively stable contact until at least the eighth to ninth centuries (Pellard, 2015), we expect more similarities between both languages than in the case that the connectivity was completely broken. Therefore, the proposed datings tend to be biased towards shallower break-up times than the real times.

Lee and Hasegawa's (2011) Bayesian analysis estimates a divergence date of Mainland Japanese and Ryukyuan (standard classification), corresponding to a divergence date of Mainland Japanese and Kyushu-Ryukyuan in the revised classification, of 2182 years ago but yields large credible intervals between 1239 and 4190 years ago (95% HPD). Even if this estimation has met with reservations (Pellard, 2015, pp. 20–21) and may be biased by several centuries towards a shallower date, a dating of the separation in the first millennium BC seems to be consistent with the spread of agriculture from Kyushu to Shikoku and Honshu.

For most of the 20th century, biological anthropologists proposed that the people of the Ryukyus – like the Ainu in the north – retained a strong genetic heritage from the Neolithic Jōmon and were less impacted by Bronze Age and later immigration into Japan (von Baelz, 1911; Hanihara, 1991; cf. Hudson et al., 2020). However, skeletal evidence for Japanese migration to the Ryukyus began to be discussed from the 1990s (Dodo et al., 1998; Pietrusewsky, 1994, 1999, 2010). Recent ancient DNA analyses have confirmed that by the seventeenth to eighteenth centuries, the genome of a peasant population on Miyako Island can be modelled as consisting of only around 20% Jōmon ancestry; genetic differences with late prehistoric people on the same island show that the new population must have arrived between AD 900 and 1600 (Robbeets et al., 2021).

The spread of Ryukyuan languages to the islands is consistent with the farming/language dispersal hypothesis (Bellwood & Renfrew, 2002) in the sense that farming and language dispersed at the same time. Yet the historical context of the medieval Ryukyus is complex and has been increasingly analysed through models emphasising trade and piracy as well as the role of neighbouring states, primarily Heian Japan, and its administrative outpost at Dazaifu in north Kyushu, as well as Silla and Koryŏ Korea. Here, we combine archaeological and linguistic data to compare the role of demographic change, trade and expanding state power on the spread of agriculture and Ryukyuan languages into the Ryukyu Islands. By evaluating the location of the Ryukyuan homeland, the break-up time of Proto-Ryukyuan, its linguistic profile and tree structure, we make inferences about the reasons why Ryukyuan languages spread to the Islands. In order to assess the different mechanisms of language spread, we apply methods of historical linguistic reconstruction. It should be noted that during the period considered in this article, the Ryukyu islanders themselves produced few written documents; the political administration of the Islands did not regularly employ writing until the sixteenth century (Smits, 2018, p. 1).

The term ‘Ryukyu Islands’ (or Ryukyus) is used here to refer to all of the islands from Tanegashima to Yonaguni. We divide the Ryukyu chain into northern (Tanegashima and Yakushima), central (Amami and Okinawa) and southern (Miyako and Yaeyama) cultural zones (Kokubu, 1966). This division deviates from the usual binary linguistic division, which classifies the Ryukyuan languages into Northern (Amami and Okinawa areas) and Southern (Miyako and Yaeyama areas), to the exclusion of the Tokara and Ōsumi islands, whose modern topolects belong to Mainland Japanese (de Boer, 2020). Linguists use the prefix ‘Proto’ for an ancestral language reconstructed on the evidence of data that are available from a later period, meaning that it is the common source of all the languages in a given family. As such, Proto-Ryukyuan (PR) is the conventional term for the ancestral language from which the contemporary and historical Ryukyuan languages descend. In addition, we use the specification Proto–Kyushu–Ryukyuan (PKR) for the presumed ancestral language of Ryukyuan and pre-medieval Kyushu dialects, which are thought to remain as a substratum in modern Kyushu dialects. The term Proto-Japonic (PJ) is used for the overall source of Japanese and all its sister languages, including Mainland, Kyushyu and Ryukyuan varieties, while Proto-Mainland Japanese (PMJ) is the Mainland branch of Japonic with the exclusion of Kyushu varieties according to the revised classification, and thus a sister branch of Proto-Kyushu–Ryukyuan.

## Methods

**Figure 3.**
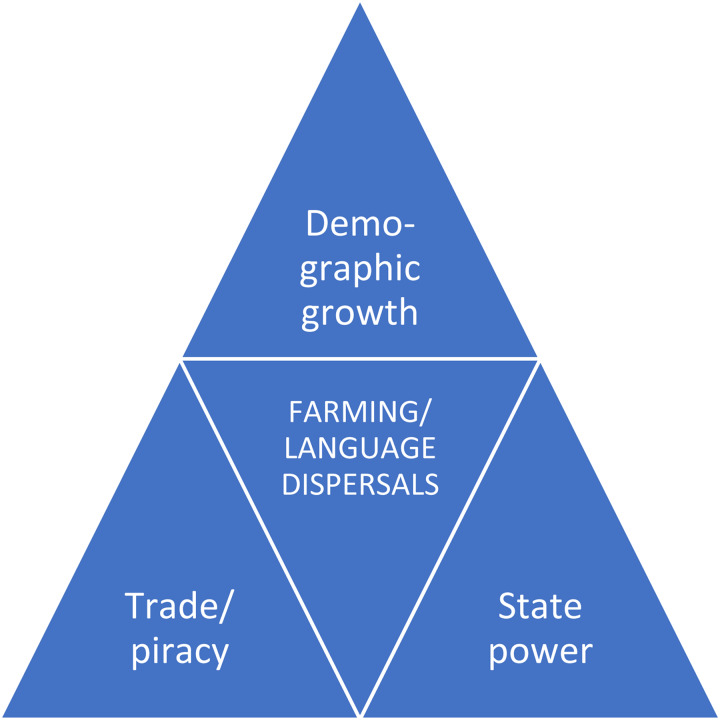
A tripartite model for farming/language dispersals to the Ryukyu Islands.

Integrating linguistic and archaeological approaches, we propose and test a tripartite model for the dispersal of farming and Ryukyuan languages which incorporates demography, trade and state power (Figure 3). These three features have been debated in previous research, but they have often been seen as competing interpretations.

Demographic growth assumes that an increase in population density led to expansions into the Ryukyus as farmers sought new land. This is the default subsistence/demography model used for the Neolithic (Renfrew, 1987; Bellwood & Renfrew, 2002). With respect to the Ryukyus, population growth may have occurred in Kyushu, or else an initial introduction of farming to one or more Ryukyu islands – perhaps through trade – may have then resulted in a secondary expansion under demographic pressure. However, sedentary peasants in Kyushu are unlikely to have packed all of their crops and belongings into boats and sailed off to the Ryukyus; any such dispersal is likely to have included specialists involved in maritime voyaging and trade.

The subsistence/demography model predicts a linguistic homeland in northern Kyushu where Yayoi farmers first settled and from where they left to Mainland Japan, leading to the separation between Proto-Mainland Japanese and Proto-Kyushu–Ryukyuan. Under this model, the expected break-up time of Proto-Kyushu–Ryukyuan would be between the eighth and thirteenth centuries AD, when agriculture started to spread to the Ryukyus. This model predicts a tree structure in which the Kyushu dialects and the Ryukyuan languages are sister clades within a Kyushu–Ryukyuan cluster, separate from Proto-Mainland Japanese. The break-away model of the Ryukyuan languages is expected to proceed from north to south in line with farming dispersals. Under this scenario, the pre-existing hunter–gatherer languages in the Ryukyus may have left some substratum interference in Ryukyuan as their speakers shifted to the language of the incoming farmers.

Trade between Kyushu and the Ryukyus from the first millennium BC onwards has been extensively analysed (Kinoshita, 1996, 2003a, 2019; Pearson, 1990, 2013). The Bronze Age shell trade led to the import of some Yayoi pottery to Amami and Okinawa but cereal cultivation was not adopted as a result. In the Middle Ages, in contrast, the spread of cereals and domesticated animals to the Ryukyus was associated with ceramic and soapstone vessels. While some scholars regard these vessels as evidence of the impact of the Japanese (Heian) state (Nakashima, 2008), most emphasise the role of merchants who were increasingly involved in trading networks around the East China Sea, networks which often attempted to bypass state controls. Trade during this period is impossible to separate from piracy and raiding (Scott, 2017; Ling et al., 2018; Smits, 2018; Hudson, 2020) and these concepts are conflated in this paper.

If the language dispersal to the Ryukyus was purely driven by trade, we might expect the development of a mixed language or creole, whereby Yayoi traders developed a common means of communication to interact with native hunter–gatherers. However, the sociolinguistic conditions for such language mixing are very specific, including the presence of a multilingual setting (as opposed to a bilingual one) and trade situations among speaker groups of more or less equal status (Thomason & Kaufman 1988). The creole would originate in trade and distribution centres where traders interacted with local populations, such as on Kikai Island (see below). Since the shell trade between Kyushu and the Ryukyus is known to have taken place from the first millennium BC onwards, language mixing is expected to have started at such an early time. As a trading creole, Proto-Ryukyuan would be very distinct from Proto-Kyushu–Japanese or its daughter clades and the Ryukyuan family would represent a multiple topology without clear correlations to Ryukyu geography. However, in this case this scenario is unlikely given that Yayoi and later traders probably enjoyed a higher social status than local hunter–gatherers. Since trade is expected to represent a model compatible with the other scenarios rather than a competing interpretation, an alternative model involving trade among other mechanisms predicts dispersal of Proto-Ryukyuan with borrowing of trade vocabulary from the local hunter–gatherer languages rather than the spread of a mixed trading language.

With respect to the role of state power, no scholars have argued that the Ryukyus were directly incorporated by the Heian state; if that were the case, the standard (administrative) Japanese used in Kyushu would presumably have been transmitted to the Islands. The question of state power has instead been approached in two ways. One is that state expansion pushed people from Kyushu to the Islands. Uemura (1977) proposed that Ryukyuan derived from a dialect spoken in southern Kyushu by the Hayato, a ‘tribal’ people who opposed the Yamato kingdom; when southern Kyushu was conquered by Yamato in the eighth century (cf. Ikehata, 1996), the Proto-Ryukyuan speakers were pushed south to the Islands. Only a few words of Hayato language are preserved in the *Ōsumi Fudoki*, an Old Japanese source from the eighth century. Although some authors have tried to connect these words to Austronesian (Kakubayashi, 1998), the mainstream idea remains that the Hayato spoke a language related to Japonic, but representing a primary break-up from other varieties spoken on Kyushu, Shikoku and Honshu (Elmer, 2019). Under this model, Proto-Ryukyuan would thus have separated from Proto-Kyushu–Japanese in the early first millennium AD at the latest and would have remained isolated in southern Kyushu until it separated and dispersed in the eighth century. The state intervention model predicts a break-away of Ryukyuan languages whereby the languages of regions that offered the highest potential for escape from state control separated first.

A second approach has been to explore the role of Dazaifu – the Heian state's administrative base on Kyushu – in establishing new outposts, most importantly on Kikai Island. As discussed below, however, the impacts of Kikai on the other islands of the Ryukyu chain are complex and contested.

In the following we first analyse the three components of demography, trade/piracy and state power from the perspective of archaeology and historical linguistics. In the Discussion the results are then combined into a single synthetic model. Without assuming that they are mutually exclusive, Table 2 lists some possible archaeological and linguistic correlations of the three approaches.

**Table 2.** Some archaeological and linguistic correlates of the components of the tripartite model for the spread of agriculture and Ryukyuan languages

	Demographic growth	Trade/piracy	State intervention
Summary of component feature	Population growth led to dispersals as farmers sought new land	The spread of agriculture and Ryukyuan was a secondary result of trade and piracy	Dispersals to the Ryukyus resulted from people escaping the state and/or from state control over some islands
Pattern of agricultural dispersal	North to south; larger, more fertile islands settled first	More complex settlement pattern centred on trading emporia	Agriculture spread first to islands under state control
Settlement continuity	More or less sudden appearance of farming settlements with new material culture	Overlap and complex interaction between late prehistoric and medieval populations	Sudden establishment of state administrative centres
Spread of associated material culture	More or less uniform spread of domestic pottery and tools from specific region such as southern Kyushu	Diverse material culture; strong links with production centres and trading emporia	Initial separation between sites within and outside state control
Expected tree structure	Proto-Kyushu–Ryukyuan vs. Proto-Mainland Japanese Proto-Ryukyuan is a sister or a subgroup of Kyushu dialects Break-away model of Ryukyuan languages from north to south	Proto-Ryukyuan vs. Proto-Kyushu–Japanese Kyushu dialects and Japanese are sister clades Break-up in line with maritime accessibility of island geography	Proto-Ryukyuan vs. Proto-Kyushu–Japanese Kyushu dialects and Japanese are sister clades Break-away model of Ryukyuan languages in line with most efficient escape of state control
Expected homeland of Proto-Ryukyuan	Northeast Kyushu	Trade centres such as Kikai Island	Southern Kyushu or Dazaifu
Expected break-up time	AD 800–1300	Wide possible range from 500 BC to AD 800	AD 800
Expected linguistic profile	Language shift from hunter–gatherer languages to Proto-Kyushu–Ryukyuan farmer language	Trading creole *or* Borrowing of trade terms in Proto-Ryukyuan	Language shift from hunter–gatherer languages to Proto-Ryukyuan ‘Hayato’ tribal language

## Results

### Archaeological approach

#### Demographic growth

Population growth in Japan slowed in the eighth century owing to smallpox and other epidemics; the years from 800 to 1150 saw demographic stasis owing to epidemics, famine and ecological degradation (Farris, 1985, 2017). A proposal made in the 1960s that there was widespread land clearance over the tenth to fourteenth centuries has been critiqued (Farris, 2009, pp. 69–73). Population growth only seems to have revived after 1300. Thus, the period when farming and Ryukyuan languages spread to the Islands was one of population stasis or decline in Japan as a whole. However, the unstable social conditions of the time often encouraged migration as peasants sought better conditions; although opposed by the court, such movements were common (Farris, 1985, pp. 118–140). As mentioned, any migration by Kyushu peasants to the Ryukyus would have required boats, which were probably controlled by persons involved in maritime trade.

The subsistence/demography model of language expansion assumes that populations with an economic advantage over their neighbours would grow in numbers and spread to new areas of settlement, leading to language shift in the latter (Bettinger & Baumhoff, 1982; Renfrew, 1987; Bellwood & Renfrew, 2002). The ecological risks of small islands mean that initial population growth would have been an adaptive response for farmers dispersing to the Ryukyus (cf. Golson, 1972; Kirch, 1984). Thus, if agriculture began on one of the Ryukyu Islands at an early stage, the population of that island may have grown quickly, forcing onward migrations.

Archaeobotanical evidence shows that agriculture moved in several distinct stages from Kyushu to the southern Ryukyus. The earliest direct radiocarbon dates on cereals from southern Kyushu are two dates on wheat ranging between AD 675 and 885 from Miyazaki (Supplementary Information 1). However, remains of rice paddy fields are known from the Early Yayoi phase (800–400 BC) from the Kagoshima University Kōrimoto Housing and Sakamoto A sites (Takamiya & Nakamura, 2021). Envoys sent by the Yamato kingdom to Tanegashima in 681 reported that ‘Rice is always abundant. With one sowing, there are two harvests’ (Aston, 1972, II, p. 352). While this suggests that rice cultivation was already common on Tanegashima by the seventh century, the official envoys may have exaggerated the fertility of the island in their report. There is little archaeological evidence for agriculture in the northern Ryukyus at this time. Analysis of impressions on 5622 sherds of Yayoi–Kofun pottery from Tanegashima found only 0.1% had cereal impressions: three rice and one foxtail millet from the Middle Yayoi and two rice seeds from the Kofun (Nakamura, 2015).

The first domesticated cereals appeared on Kikai in the Amami archipelago in the eighth century and Okinawa in the tenth century, suggesting a delay of a century or two between the two archipelagos. Sites dating to before the medieval era in these islands have produced only wild plants, except for bottle gourd from the Ireibaru site. Early agriculture in the Amami archipelago was characterised by a diversity of crops. In Okinawa, in contrast, foxtail millet was the dominant crop at the beginning of the medieval period, but by the thirteenth century some sites have a high proportion of wheat. Rice and barley are also known. Rice paddy fields have been confirmed from the beginning of the medieval era at sites such as Maeatari and Aragusuku Shichibaru No. 2 (A. Shinzato, 2018a, b). In the southern Ryukyus, soil flotation has so far only been conducted at three sites on Miyako where the Neolithic Arafu and Nagabaka sites yielded no cultigens (Takamiya, 2003; Robbeets et al., 2021), while at medieval Minuzuma over 300 carbonised seeds were recovered, including foxtail millet (109), barley (90), wheat (31), *mugi*, i.e. barely or wheat (53), Fabaceae, possibly adzuki group (16), and rice (6) (Chida, 2015; Manabe et al., 2019). Radiocarbon dates place these remains between the thirteenth and fourteenth centuries.

**Figure 4.**
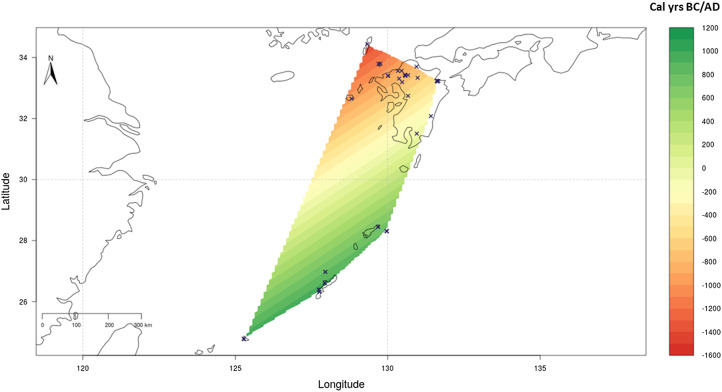
Bayesian chronological estimates of first cereal appearance across Kyushu and the Ryukyus. Estimates made using direct radiocarbon dates on cereal remains (Supplementary Information 1), modelled using the Bayesian model SpreadR (Cubas et al., 2020).

We used the Bayesian model SpreadR developed within the Pandora and IsoMemo initiatives (https://www.isomemoapp.com) to estimate the first appearance of cereals across the southern Japanese islands (Figure 4). Chronological modelling was done on a compilation of 69 direct radiocarbon measurements from barley, millet, rice and wheat grains extracted from Robbeets et al. (2021) and included here as Supplementary Information 1.

Cattle, horses, goats and chickens seem to have been introduced to the Ryukyus at the same time as cereals, although the archaeological evidence is not yet sufficient to map routes and timings. As in Japan, the status of pigs vs. wild boar is controversial. While age profiles suggest active management of *Sus* populations, domesticated pigs can rarely be distinguished on skeletal morphology (Toizumi, 2014, 2018). With the arrival of agriculture, the medieval era saw a decline in fish consumption in the Ryukyus (Toizumi, 2014, pp. 72–73).

**Figure 5.**
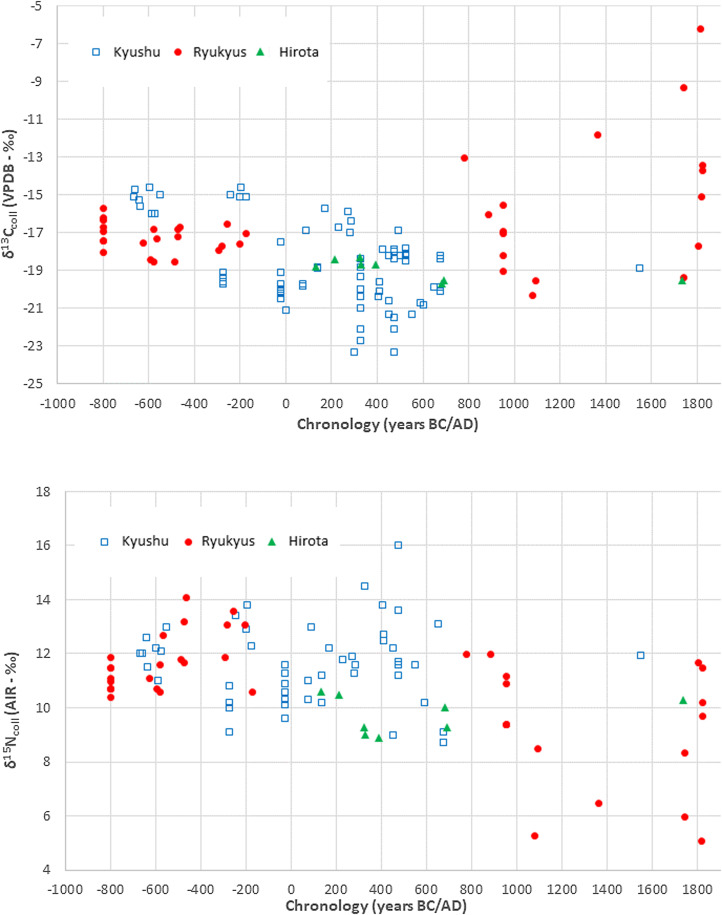
*δ*^13^C_coll_ (top) and *δ*^15^N_coll_ (bottom) values from human bone collagen from Kyushu and the Ryukyus, ca. 1000 BC to AD 1900. Data from Fernandes et al. (2021) with the date of the isotopic points set as the median of the chronological range given in the publication.

Ishidō (2014) argued that stable carbon and nitrogen isotope values (*δ*^13^C_coll_ and *δ*^15^N_coll_) for human bone collagen samples from the Yayoi–Kofun Hirota site on Tanegashima were similar to those of contemporary populations in Kyushu, assumed to be rice farmers. However, faunal remains from Layer VI at Hirota, dating to the final Yayoi to earliest Kofun, include wild boar, deer, monkeys, sea turtle and fish and shellfish (Toizumi, 2007). Using the ARCHIPELAGO isotope database for Japan (Fernandes et al., 2021), we plotted bone collagen isotopic values for Bronze Age to early modern sites from Kyushu and the Ryukyus (Figure 5).

Compared with Kyushu samples from the same time period, *δ*^15^N_coll_ values from Hirota are clearly lower and indicate a comparatively higher consumption of plant protein. In contrast, *δ*^13^C_coll_ values for Hirota are close to expected values (c. −20‰) for a C_3_-type diet (e.g. nuts, cereals such as barley or rice, and animals that consume most plants in temperate zones) or just above this, which could indicate minor contributions from marine protein and/or C_4_ plants (e.g. millet). High *δ*^15^N_coll_ and *δ*^13^C_coll_ values observed for Kyushu are typical of marine protein consumers with some individual variability. However, some Kyushu individuals also show *δ*^13^C_coll_ values below typical C_3_ references and this includes three Initial Jōmon samples from Iwashita (Nagasaki) with rather low *δ*^15^N_coll_ values (6–7‰) typical of exclusive plant diets which would exclude consumption of freshwater protein. Although the *δ*^13^C_coll_ signal is primarily determined by dietary protein, it does include a significant contribution from carbohydrates and lipids (Fernandes et al., 2012). Food fats have significantly lower *δ*^13^C values than protein and it is thus likely that some Kyushu individuals with comparatively low *δ*^13^C_coll_ values were consuming high amounts of animal fats and/or fat-rich plants (Fernandes et al., 2012).

Around AD 1000 there is a decrease in *δ*^15^N_coll_ values for the Ryukyus with values similar to those for Hirota, or even lower, suggesting an increased dependence on plant foods and lower consumption of marine protein. This is supported by zooarchaeological evidence showing that fish consumption declined with the introduction of farming. Later periods show considerable isotopic variability among individuals including large differences in the consumption of millet and animal protein.

Minagawa et al. (2005, p. 100) mention that two *Sus* bone samples from the Gushibaru site (Ie Island), to which they assign dates of ‘1800–1700 BP’, ‘are characterized by an extremely high ^13^C content, suggesting that they fed [on] mainly C_4_ plants as their staple diet. Since no similar values were found for sites in Honshu and Kyushu, these *Sus* might have been transported from areas where farming of C_4_ millet was already established.’ Northern China is suggested as a possibility by Minagawa and colleagues. Even if a few pigs were introduced from China at this time, however, the more direct archaeobotanical record shows no evidence of millet or other farming in the Ryukyus at this date. Furthermore, the Gushibaru samples have not been directly dated and may derive from later contexts.

#### Trade/piracy

Trade between Kyushu and the Ryukyus was a key component in farming and language dispersals. While some scholars have suggested that the Bronze Age shell trade brought farming to the Islands (Takamiya, 1985), the archaeobotanical record provides no evidence for such a link. According to Nakazono (2011), a ‘significant amount’ of Yayoi pottery was transported to Okinawa from Kyushu at the height of the shell trade, but in the following Kofun period imported pottery was rare. A graph by Takayuki Shinzato (2018, p. 33) shows almost 90 Yayoi vessels found in the Okinawa archipelago; typologically, these pots date from the end of the Early to the first half of the Middle Yayoi (c. 450–150 BC). Most of this Yayoi pottery originated on the Satsuma peninsula and comprised more storage jars than cooking vessels. Only one Okinawan pot has been found on Kyushu, at the Takahashi site, a settlement which Nakazono (2011) regards as a major entrepôt in the shell trade. The Yayoi shell trade involved rather limited socio-economic exchange between Okinawa and Kyushu with little lasting settlement in the Islands (Hudson in press-a).

In the medieval era, economic relations between the Ryukyus and Kyushu were transformed when cereal agriculture, iron tools, spindle whorls and new food vessels spread south. While the Ryukyus might be considered marginal to the late Heian state based in Kyoto, the artefacts associated with the spread of farming and Ryukyuan languages were international and associated with urban contexts in Kyushu. Three main types of food vessels reached the Ryukyus at this time: soapstone (steatite) cauldrons, *kamuiyaki* stoneware and Chinese trade ceramics (Pearson, 2007, 2013; A. Shinzato, 2014, 2018a). Produced in Nagasaki, soapstone cauldrons with lug handles had a distribution centred on Hakata and Dazaifu as well as Kikai. *Kamuiyaki* is a grey stoneware produced on Tokunoshima Island from the eleventh century using kiln technology derived from Koryŏ Korea and Japan (A. Shinzato, in press). As noted by Pearson (2007, p. 123), the *kamuiyaki* kilns raise numerous questions as to why stoneware production was suddenly begun on a remote island, where the technology came from and who financed the production. Chinese ceramics, especially Song whitewares, became widely distributed in the Ryukyus at this time.

Asato (1990, 1998) has suggested that merchants based in Hakata played a key role in trading soapstone vessels, *kamuiyaki* and Chinese ceramics to the Ryukyus in exchange for turban shells (*Turbo marmoratus*, used for mother-of-pearl inlay), sulphur and wood from *Bischofia javanica* (*J. akagi*, or ‘red tree’, used to produce red dye). By the twelfth century, these three types of vessels were also found in the southern Ryukyus, which for the first time became part of the same cultural zone as Okinawa and Amami, suggesting that the search for trade goods was expanding ever further south (Kinoshita, 2003b). Pearson (2013, p. 149) argues that the ultimate cause of these changes was growing commercialisation in Song China, what Elvin (1973) called the ‘medieval economic revolution’.

It has been proposed that Kikai Island served as a base for Dazaifu or other state-related persons from the ninth to early eleventh centuries (Nakashima, 2008). Excavations at the Gusuku sites group on Kikai have produced remains of buildings including storehouses, iron-working hearths and soapstone cauldrons. Local Kaneku earthenware is rare, with pottery from Kyushu as well as China and Koryŏ Korea dominating the ceramic assemblage (Pearson, 2013, pp. 158–165; Smits, 2018, pp. 20–21). Interestingly, *Sue* ware sherds from the Nakadake kiln cluster (Kagoshima) have been found at the Gusuku sites on Kikai and on Tanegashima (Sterba, 2015). This *Sue* production centre was not established near the provincial administration in northern Satsuma as might be expected, but was further south, on the estuary of the Manose river near the above-mentioned Takahashi site, which was a trade hub with the Ryukyus from the Bronze Age shell trade to the Middle Ages. The estuary had also been the centre of a powerful Hayato clan until its subjugation in the early eighth century (Nakamura & Shinoto, 2015; Nakamura, in press). The location of the Nakadake kilns shows the beginning of fundamental changes in the southern periphery of the Japanese state, where the indigenous Hayato and their local elites were in competition with the central authorities and trade and seafaring began to play the important role that they fully adopted in the Middle Ages.

Dazaifu's main interest in Kikai was probably as a place to control trade in goods such as shells, sulphur and *Bischofia javanica*. Given that texts such as the *Shōyūki* mention captives taken in raids, slaves must also be considered in this context. Settlement of Kikai at this time led to the early introduction of agriculture and – based on an increase in the number of sites – population growth on a small (57 km^2^) island (Asato, 2013). While Takanashi (2005, p. 210) emphasises the role of trade in further dispersals south of Kikai, Asato (2013) argues that population growth was the key factor. The Gusuku sites complex seems to have begun in the ninth century as a trading outpost of Dazaifu, suggesting a link with the ‘state intervention’ discussed in the next section. By the eleventh century, however, Kikai Island had shifted away from Dazaifu control and had become a centre for non-state trade (Nakashima, 2008).

#### State intervention

The ancient Japanese state relied on agriculture – especially alluvial rice farming – to finance its fiscal basis. As the state bureaucracy became more structured during the eighth century, it attempted to transform other resources into what Scott (2009, p. 73) calls ‘state accessible products’ or SAPs. Routes of maritime transport were given ritual protection by sites such as those on Okinoshima Island between Kyushu and Korea. However, the ability of ancient Japan to make ‘state space’ of the sea – again the term is from James Scott – was limited (Hudson, 2016, in press-b). The maritime spaces around Japan were increasingly utilised by non-state actors including merchants and pirates. States require the acquisition and control of people: ‘The imperative of collecting people, settling them close to the core of power, holding them there, and having them produce a surplus in excess of their own needs animates much of early statecraft’ (Scott, 2017, p. 151). Forced re-settlement was a common measure used by early states (Korolkov & Hein, 2021). The ancient Japanese state was no exception. Border guards (*sakimori*) sent to northern Kyushu were brought from the Kantō region (Farris, 1995, pp. 54–55). Refugees from Korea were sent to eastern Japan on two occasions in 870 alone (Batten, 2006, p. 89). In southern Kyushu, the above-mentioned ‘Hayato’, who had previously opposed the state, were used as ‘ethnic soldiers’ in the Kinai capital region (Hudson, 1999, pp. 194–197).

Some ‘Hayato’ (broadly understood) living in southern Kyushu may have escaped state control and moved south to the Islands, as suggested by Uemura (1977). Such people might have established new farming settlements in the Ryukyus but many probably joined pirate bands operating in the islands and coasts around western Kyushu. Groups from Amami – called *namban* or ‘southern barbarians’ by the state – attacked Kyushu, capturing 400 people from Ōsumi in 996 and plundering Dazaifu in 997 (Smits, 2018, pp. 18–20). Such groups probably included women, some of whom were pirates in their own right (Shapinsky, 2014, pp. 146–150). In 998, Dazaifu ordered Kikai to suppress the Amami pirates but it is not known what measures, if any, were actually attempted (Smits, 2018, pp. 18–20).

Even if Kikai Island seems to have been under the control of Dazaifu from the ninth to early eleventh centuries, this does not necessarily mean Dazaifu controlled all trade with the Ryukyus. The Song dynasty flooded East Asia with large quantities of export ceramics to increase its revenues (Yamamoto, 2008, p. 5) and the role of Dazaifu and the Heian state in that trade may have been quite minor. It can further be assumed that ‘pirate’ activities continued, as demonstrated by the *namban* attacks from Amami.

### Linguistic approach

#### Linguistic inferences about the most probable tree structure

As noted, the classical view in linguistics is that Japonic languages can be divided into two subgroups, Mainland and Ryukyuan, with a sharp divide between the two falling between the Tokara (linguistically Mainland) and Amami (linguistically Ryukyuan) Islands (Katō, 1977; Lee & Hasegawa, 2011; Pellard, 2015; Robbeets et al., 2021). This view has been challenged by a classification of up to four subgroups, one of which is the Kyushu–Ryukyuan ancestor of Ryukyuan (Unger, 2009, p. 105; Igarashi 2017, 2018; de Boer, 2020, pp. 56–57; cf. Figure 2). Contacts and, as a result, intensive borrowing between Mainland Japanese and Kyushu–Ryukyuan can be assumed to have continued after the separation of these two groups. Linguistic evidence for Early Middle Chinese (EMC) (AD 600–900) loanwords being transmitted from Early Middle Japanese (EMJ) (AD 800–1200) to Proto-Ryukyuan (PR) and having regularly corresponding reflexes in all Ryukyuan languages (e.g. EMC *baɨwŋʰ* ‘stick’ >> EMJ *baũ* >> PR *bau in Shuri (Okinawa) *bóː*, Ogami (Miyako) *pau* and Dunan (Yonaguni) *bûː*) suggests that these contacts continued until at least the ninth century (Pellard, 2015). After the separation of Ryukyuan from Kyushu–Ryukyan, small groups of Ryukyuan speakers dispersed over the Ryukyu Islands. A founder effect caused an increase in the speed and extent of differentiation from the proto-language, while the Kyushu speakers remaining in Kyushu continued their close interactions with Mainland Japanese speakers and eventually shifted to Mainland Japanese.

In accordance with the maximum parsimony method of classical historical linguistics, which seeks a classification that explains a dataset by minimising the number of evolutionary changes required, evidence for the genealogical clustering of Ryukyuan languages with Kyushu topolects is based on shared innovations in vocabulary, phonology and morphosyntax between both groups (Supplementary Information 2). The agricultural vocabulary of Proto-Kyushu–Ryukyuan is largely continuous with that of its Proto-Japonic predecessor, even if there are a few common innovations shared by Kyushu and Ryukyuan topolects only. This confirms that the Proto-Kyushu–Ryukyuan speakers were farmers who inherited agriculture from their Japonic ancestors. Compared with other areas of subsistence, it is seafood-related vocabulary that reveals most copiously shared traits between Kyushu and Ryukyuan as opposed to other Japonic topolects. This is the case in spite of the fact that Kyushu and the Ryukyus belong to different climate zones with different marine fauna. The proportion of innovations in the shared Kyushu–Ryukyuan vocabulary in this semantic domain permits the conclusion that the Kyushu–Ryukyuan farmers developed a more maritime, seafaring culture after the break-up of Proto-Japonic.

As well as lexicon (Igarashi, 2017, 2018; Jarosz, 2019), we find numerous shared innovations in phonology and morphosyntax between Kyushu topolects and Ryukyuan languages. Some of these properties, like case marking, have a broad Kyushu distribution, while others, such as the use of the interrogative marker *-*na* in yes/no questions, appear limited to southern Kyushu and its outlier islands such as Kami–Koshiki. This observation supports the classification of Proto-Kyushu–Ryukyuan as a branch separate from Proto-Mainland Japanese and suggests that Proto-Ryukyuan originated from a subgroup of southern Kyushu dialects as proposed by de Boer, Unger and Igarashi. The resulting classification is in line with the demography model and contradicts the development of a separate trading creole in the Ryukyus. It also goes against the conception of Proto-Ryukyuan as the language spoken by the ‘Hayato’, isolated from other Proto-Kyushu–Japanese speakers. Nevertheless, the classification leaves room for simultaneous maritime interactions with traders or a parallel intention to escape state control to be integrated in the model.

Both classical views and Kyushu–Ryukyuan classifications converge on the break-up of Proto-Ryukyuan into northern and southern branches. This binary structure is less consistent with the gradual north to south spread of agriculture across the Ryukyus and also does not suggest the inverse, namely that southern islands which escaped state control more effectively than northern islands were colonised first. The primary break-up between northern and southern languages rather seems to align with island geography, reflecting the maritime boundary between the northern and southern islands. Therefore, it seems to be more consistent with maritime accessibility, which is a prerequisite for trade.

#### Linguistic inferences about the most probable homeland of Proto-Ryukyuan

The Kyushu area has long been pinpointed as the most likely homeland of Proto-Ryukyuan (Hattori, 1976a; Thorpe, 1983; Uemura, 1997; Serafim, 2003; Pellard, 2015; Karimata, 2017; Igarashi, 2018), but the exact location on Kyushu is a matter of disagreement. Within centuries after the migration, the Proto-Kyushu–Ryukyuan population remaining in Kyushu abandoned their own language and shifted to Mainland Japonic – a likely sociolinguistic outcome of the expansion of the power of the Mainland-speaking Japanese state across Kyushu.

Given the shift from Proto-Kyushu–Ryukyuan daughter language(s) to Mainland Japonic on Kyushu, a substratum of the abandoned Kyushu–Ryukyuan language(s) might be expected to be found in modern Mainland Kyushu topolects. Moreover, those Kyushu areas whose topolects have the most evidence of such a substratum can be hypothesised as the most likely candidates for the starting point of linguistic dispersal into the Ryukyus and thus the homeland of Proto-Kyushu–Ryukyuan. A study of non-basic vocabulary (Jarosz, 2019) evidenced that the thickest layer of vocabulary was shared between Ryukyuan and the Kagoshima and Morokata areas in southern Kyushu, referred to as the Satsugū area in Japanese dialectology, and especially with outlier islands such as Tanegashima, Yakushima, Tokara and Koshiki. Moreover, in the revised division of Japonic into Kyushu–Ryukyuan and Mainland Japanese branches proposed by de Boer (2020) and Igarashi (2017, 2018), the southernmost unit centred in the Kagoshima area was identified as the subunit from which Ryukyuan branched off. We therefore infer that the most plausible homeland for Proto-Kyushu–Ryukyuan is in southern Kyushu and neighbouring islands such as Tanegashima, Yakushima, Tokara and Koshiki. Bayesian phylogeographic modelling (Robbeets et al., 2021) also supports southern Kyushu as the most probable point from where the linguistic dispersals to the Ryukyus took place. If Proto-Kyushu–Ryukyuan indeed constitutes a valid genealogical unit, the hotspot where we find the greatest linguistic diversity with regard to the Kyushu and Ryukyuan sub-branches would be located on the interface between the Tokara and Amami Islands, and Kikai Island would thus be a plausible homeland for Proto-Ryukyuan.

These findings contradict Serafim's (2003, p. 471) proposal that ‘if any specific Kyushu dialect is a later form of the dialect from which Ryukyuan descends, it is the dialect in the northeastern part of Kyushu adjacent to the strait opposite from Yamaguchi prefecture’. While such a northern location of the Proto-Kyushu–Ryukyuan homeland might be in line with a demic diffusion of agriculture, a southern location is more consistent with the trade and state intervention models, although this does not exclude that agriculture was involved as well.

#### Linguistic inferences about the most probable break-up time of Proto-Kyushu–Ryukyuan

Most linguists agree that Proto-Ryukyuan was spoken on Kyushu for some time until its speakers moved southward to settle the Ryukyus. In the traditional classification, the first break-up in the Ryukyuan family is estimated at around AD 1300 (Robbeets et al., 2021) or 1000 (Lee & Hasegawa, 2011) and is reminiscent of the break-up time between Ryukyuan and Kyushu Japanese of around the tenth century AD suggested by de Boer (in press) and Unger (2009, p. 100, 104–105). As previous research has linked the spread of farming to the Ryukyu Islands with the spread of new populations and Ryukyuan languages between the eighth and thirteenth centuries AD (Hudson, 1994, 1999; Pellard, 2015; Takamiya, 2004), the dating of the break-up between Ryukyuan and Kyushu Japanese seems to be consistent with agricultural spread. Although this spread postdates the conquest of southern Kyushu by Yamato, which can be dated to the time of the subjugation of the last uprising in southern Kyushu in 720–721 and the 740 rebellion by Fujiwara no Hirotsugu (Nagayama, 2009, pp. 80–95), it does not completely exclude the state intervention model. The dating does not coincide with the beginnings of the shell trade between Kyushu and the Ryukyus, since such relations are known to have existed from the first millennium BC. Yet it is nevertheless consistent with the new trade patterns with the Ryukyus which developed from the eighth century AD.

#### Inferences about the most probable linguistic outcome of the migration

Although most of the shared Kyushu–Ryukyu innovations discussed above may be due to broken connectivity with Mainland Japanese followed by steady and continuous internal language change, some properties may have been carried over from a non-Japonic (Jōmon) linguistic substratum. This is especially true for changes against the grain of the language, such as the increased frequency of voicing in initial position, which goes against a proto-typical Transeurasian characteristic. Even if in a situation of language shift general phonological features are expected to be carried over more easily than concrete lexemes (Thomason & Kaufman, 1988), some of the shared maritime vocabulary, lacking cognates elsewhere in Japonic, may also have been coined under influence of this Jōmon substratum.

Given the low proportions (~10%) of the Jōmon genome in the Yayoi population of Kyushu (Robbeets et al., 2021), we can assume that local hunter–gatherers became absorbed by Yayoi farmers, from whom they adopted agriculture and language, although some groups may have developed specialised fishing and hunting adaptations even while experiencing gene flow from farming populations (Hudson, 2019, in press-b). After the dispersal of Mainland Japonic from Kyushu to Honshu and Shikoku around 200 BC, the remaining Kyushu–Ryukyuan speakers spread across Kyushu from the north to the southwest, where they further intermarried with local Jōmon populations. The hunter–gatherers in the south abandoned their own language, but not without leaving some phonological traces in the newly acquired language and coining maritime vocabulary that was missing from the target language. It is from this location in southern Kyushu that the Ryukyuan speakers eventually left to spread agriculture and language down the Ryukyu Islands around AD 1000.

The farming/language dispersal scenario, whereby pre-existing hunter–gatherers shift to the language of the incoming farmers, is consistent with the demography component in Figure 3. On the other hand, the lack of a detectable non-Japonic substratum in the vocabulary for trade goods from before the twelfth century, such as sulphur or turban shells (Supplementary Information 2), contradicts the prediction about the origins of Proto-Ryukyuan as a trading creole.

## Discussion

In terms of archaeology, the following conclusions with respect to the features in Table 2 are supported by our analysis. First, the pattern of agricultural dispersal into the Ryukyus, while generally following north to south increments, also includes early settlement of small islands such as Kikai. Given the complex history of Kikai, this pattern suggests trade and state control were significant factors in language/farming dispersals. The fact that there is little evidence for settlement overlap between native hunter–gatherers and incoming farmers suggests a rapid advance of agricultural populations within each context. For the third factor of the spread of material culture associated with farming, there is little evidence that agriculture spread with a set of domestic ceramics and tools for cultivation – as was often the case in the Neolithic (cf. Shennan, 2018). From the eleventh century, locally made earthenware in the Ryukyus known as Gusuku type pottery displays strong similarities with medieval pottery from Japan (Pearson, 2013, p. 150). Yet the ceramic assemblage of the medieval Ryukyus is dominated by Chinese and Korean trade wares; plain domestic earthenware is considerably less common (A. Shinzato, 2018b, p. 162). The spread of agriculture down the Ryukyus in association with these high-quality trade wares does not support a ‘wave of advance’ of peasant farmers in search of land. Few farming tools have been discovered from early medieval Ryukyu sites. However, iron-working features have been excavated from the twelfth century on Kikai and it seems likely that sickles and other iron tools were introduced with farming.

In order to determine which of the three components in Figure 3 is most consistent with the linguistic evidence, Table 3 evaluates different predictions with regard to the tree structure, location of the homeland, age and linguistic profile of Kyushu–Ryukyuan. We find that the demography model is best supported by the data because the structure of the Japonic tree including a Kyushu–Ryukyuan branch, the dispersal time of Ryukyuan estimated around AD 800–1300 and the etymological profile indicating language shift from a non-Japonic fishermen's language to the Proto-Kyushu–Ryukyuan farmer language are consistent with the farming/language dispersal hypothesis. Nevertheless, some of our linguistic findings suggest that demography does not necessarily exclude other motivations such as trade and escape from state control. The primary break-up of the Ryukyuan family into northern and southern sub-branches, for instance, may mirror island geography and accessibility for maritime trade. Finally, the assumption that the Ryukyuan language separated and dispersed from a common ancestor situated in southern Kyushu indicates that escape from state control may also have been a factor in the dispersal of Ryukyuan.

**Table 3.** Testing linguistic correlates of the components of the tripartite model for the spread of the Ryukyuan languages. Expectations matching the linguistic findings are marked in bold

	Expected if DEMOGRAPHY	Expected if TRADE	Expected if STATE CONTROL	FINDINGS
Tree structure	**Proto-Kyushyu-Ryukyuan vs. Proto-Mainland-Japanese** **Proto-Ryukyuan is a sister or a subgroup of Kyushu dialects** Break-away model of Ryukyuan languages from north to south	Proto- Ryukyuan vs. Proto-Kyushu–Japanese Kyushu dialects and Japanese are sister clades **Break-up in line with maritime accessibility of Island geography**	Proto-Ryukyuan vs. Proto-Kyushu–Japanese Kyushu dialects and Japanese are sister clades Break-away model of Ryukyuan languages in line with most efficient escape of state control	**Proto-Kyushyu-Ryukyuan vs. Proto-Mainland-Japanese** **Proto-Ryukyuan is a subgroup of southern Kyushu dialects** **Primary break-up between Northern and Southern Ryukyuan languages**
Homeland of Proto-Ryukyuan	Northeast Kyushu	Trade centres such as Kikai Island	**Southern Kyushu**	**Southern Kyushu**
Break-up time	**AD 800–1300**	Ca. 500 BC to AD 800	AD 800	**AD 800–1300**
Linguistic profile	**Language shift from hunter–gatherer languages to Proto-Kyushu–Ryukyuan farmer language**	Trading creole *or* Borrowing of trade terms in Proto-Ryukyuan	Language shift from hunter–gatherer languages to Proto-Ryukyuan ‘Hayato’ tribal language	**Agricultural cognates between Proto-Mainland -Japanese and Proto-Kyushu–Ryukyuan** **Possible substratum inference of a non-Japonic fishermen's language**

Like the three aspects of a crime that must be established to prove guilt, language spread usually involves opportunity, means and motive (Robbeets, 2017). The *opportunity* has to do with the conditions of time and space in which the ancestral language is situated and over which the ancestral speakers have little or no control. Escape from state control may be such a push-factor, inviting speakers to spread. The *means* refers to the force, instrument or technology that drives the spread. Advantages in maritime transport or economic considerations relating to trade may empower speech communities to spread and to interact with other communities. Finally, language dispersal also requires a *motive*, a mechanism that causes the dispersal, such as demographic growth. Through considering these three aspects, we can reach a tripartite model for language spread in which the different accounts complement each other.

## Conclusions

Our interdisciplinary analysis supports a link between the spread of agriculture and the Ryukyuan languages. Recent DNA analysis shows that large-scale migration from Japan into the Ryukyus had occurred by the early modern era (Robbeets et al., 2021), a migration which most likely occurred in association with the medieval dispersal of farming and Ryukyuan languages. We conclude that demographic growth, trade/piracy and the expansion of Japan's Heian state were all significant factors in these dispersals. However, the role of these factors changed over time. Heian control of Kikai Island, for example, seems to have only lasted a few centuries. Our analysis of the probable homeland of Proto-Ryukyuan concludes that southern Kyushu and neighbouring islands in the northern Ryukyus is the most likely location.

## Data Availability

Data used in this paper can be found in the supplementary information, the cited references and in the ARCHIPELAGO database (https://pandoradata.earth/dataset/archipelago-human-stable-isotope-database).

## Supplementary material

For supplementary material accompanying this paper visit https://doi.org/10.1017/ehs.2022.1. click here to view supplementary material
