# Supplementary material for: Demography, trade and state power: a tripartite model of medieval farming/language dispersals in the Ryukyu Islands
Source: Evol Hum Sci. 2022 Jan 26;4:e4. doi: 10.1017/ehs.2022.1 (PMC10426105; doi:10.1017/ehs.2022.1)
Supplement: Supplementary file 1 [file S2513843X22000019sup.zip › S2513843X22000019sup002.docx]

**Aleksandra Jarosz *et al*., ‘Demography, trade and state power: a tripartite model of medieval farming/language dispersals in the Ryukyu Islands’.**

**Supplementary Information 2**

*Linguistic inferences about the most probable tree structure*

The classical view in Japonic linguistics (Katō, 1977; Lee & Hasegawa, 2011; Pellard, 2015; Robbeets et al., 2021) that the Japonic languages can be divided into two subgroups, Mainland and Ryukyuan, with a sharp divide between the two falling between the Tokara (linguistically Mainland) and Amami (linguistically Ryukyuan) islands, has recently been challenged by a classification of up to four subgroups, one of which is the Kyushu-Ryukyuan ancestor of Ryukyuan (Unger, 2009, p. 105; Igarashi 2017, 2018; de Boer, 2020, pp. 56-57; see Fig. 2 in the main text).

It is assumed that at a time after the separation of Mainland Japanese, contacts between the two groups continued, resulting in intensive borrowing between Mainland-Japanese and Kyushu-Ryukyuan. Linguistic evidence for Early Middle Chinese (EMC) (AD 600-900) loanwords being transmitted to Early Middle Japanese (EMJ) (AD 800-1200) and to Proto-Ryukyuan (PR) and having regularly corresponding reflexes in all Ryukyuan languages (e.g., EMC *baɨwŋʰ* ‘stick’ >> EMJ *baũ* >> PR **bau* in Shuri (Okinawa) *bóː*, Ogami (Miyako) *pau* and Dunan (Yonaguni) *bûː*) suggests that these contacts continued until at least the 9th century AD (Pellard, 2015). After the separation of Ryukyuan from Kyushu-Ryukyuan, small groups of Ryukyuan speakers dispersed over the different Ryukyu Islands, with a founder effect causing an increase in differentiation from the parent speakers from which they derived, while the Kyushu speakers remaining in Kyushu continued their close interactions with Mainland Japanese speakers and eventually shifted to Mainland Japanese.

In accordance with the maximum parsimony method of classical historical linguistics, which seeks a classification that explains a dataset by minimising the number of evolutionary changes required, evidence for the genealogical clustering of Ryukyuan languages with Kyushu topolects is based on the following shared innovations between both groups.

*1. Shared innovations in vocabulary*

Previously, linguists have proposed over 70 lexical items shared between the Ryukyuan languages and the Kyushu topolects that are not present in Mainland Japanese (Igarashi, 2017; de Boer, 2020, p. 55; Jarosz, 2019).^[[1]](#footnote-1)^ Even if a good number of these may concern shared retentions, there are also clear examples of shared innovations, such as PKR **tubusi* ‘knee’, a possible replacement of PJ **pinsa* ‘knee’, **pum*- ‘to wear shoes’, which developed from PJ **pum*- ‘to tread’, **suba* ‘lips’ and **ado* ‘heel’.

When considering the agricultural vocabulary in particular, our Supplementary Information 3 shows that the bulk is shared between Ryukyuan, Kyushu and Mainland Japonic varieties. This list is composed by adding maritime vocabulary and cognates from Kyushu topolects to the Japonic agricultural vocabulary compiled in Robbeets et al. (2021: Supplementary Data 1). It includes crop names such as ‘barley’, ‘bean’, ‘broomcorn millet’, ‘foxtail millet’, ‘hemp’ and ‘potato (tuber)’, parts or properties of cultivated plants, such as ‘bran’, ‘cereal ear’, ‘threshed grain’, ‘seed’, ‘straw’ and ‘glutinous (of a cereal)’, terms relating to instruments and facilities for cultivation such ‘plough’, ‘cultivated (dry) field’, ‘rice field’, ‘storehouse’ and ‘straw bag’, agricultural practices such as ‘to harvest’, ‘to hull’, ‘to plant’, ‘to ripen’ and ‘to sow’, and words relating to food production such as ‘flour’, ‘malt, yeast’, ‘cooked cereal meal’, ‘soup/broth’ and ‘to grind’. All of these words go back to common Proto-Japonic origins.

In addition, we find a number of items shared between Kyushu topolects and Ryukyuan languages that are missing from Mainland Japanese. Some of these are shared retentions, such as the word for ‘agricultural field’ (SI 3: row 249), PKR **paru* with cognates found in Kagoshima *hai* ~ *haru*, Morokata and Ōita *haru*, Old Shuri *haru*, Yoron *paru*, Yonamine *paruu*, Tarama *paru*, Hiroma *pari*, Ikema *hai*, Sesoko *faruː* and Yonaguni *haru.* This word is a retention of PJ **paru* with the same meaning, a deverbal noun from PJ **paru*- ‘to open, clear’, reflected in among others OJ *par*- ‘open new ground, clear land’ and OJ *paruk*- ‘open up, clear up, get bright’ and a deverbal noun with vowel assimilation OJ *para* ‘field, plain, prairie’ (Robbeets, 2005, p. 106).

Others are shared innovations, such as ‘non-glutinous (rice)’ (SI 3: row 608), PKR **saku* attested in Kagoshima *saggome*, Koshiki *saːku* ~ *saggome* ~ *sakumai* ‘non-gluttinous rice’, Tanegashima *sakumaː* and Miyazaki *ɕaku*, Hiroma *safuni* ~ *safunimaz* ‘non-gluttinous rice’, which may be derived from a verb PKR **sak*- ‘to ferment, bloom’ followed by **kome* or **mapi* ‘rice’.

The latter term for ‘rice’ (SI 3: rows 597, 599) is another PKR innovation. Although **mapi* does not have unambiguous cognates in Mainland Japonic, it could be related to Old Japanese *mapi_1_* ‘gift, offering’, which may be a deverbal noun from PJ **map*- ‘to offer’, underlying in OJ *tamap*- ‘to give’ and *maukar*- ‘to profit’. The Kyushu attestations of **mapi* are restricted to two insular South Kyushu topolects, Tanegashima *sakumaː* and Kami-Koshiki *sakumai*, either case being a compound. On the other hand, in Ryukyuan languages **mapi* is broadly attested as a standalone lexeme, which in most South Ryukyuan languages as well as some North Okinawan/South Amami varieties indicates ‘rice grain’, ‘rice plant’ and ‘cooked rice’: cf. Yoron *mai*, Sesoko *meː*, Ikema *mai*, Hiroma/ Nagahama/ Ogami/ Tarama *maz*, Ishigaki *mai*, Hatoma *mai* ‘rice, rice plant’, Hateruma *məː* < **maɨ*, Kohama/ Taketomi *mai* and Yonaguni *mai*.

Ryukyuan and Kyushu also share names for a number of tools and containers, such as ‘gimlet, hand drill, awl’ (SI 3: row 998), ‘straw bag’ (SI 3: row 811) and ‘winnowing basket for separating chaff from grain’ (SI 3: row 960). However, PKR **iri* is a likely development from PJ **kiri* ‘hand drill, awl’ reflected in OJ *ki_1_ri* ‘awl, pointed tool for piercing small holes’, which is in turn a deverbal noun from PJ **kir*- ‘to cut’, reflected in OJ *ki_1_r-* ‘to cut, shear, chop’. The word for ‘straw bag’, PKR **kamage* is probably a derivation from PKR **kama* ‘reed’, which goes back to PJ **kama* ‘reed’, reflected in OJ *kama* ‘reed, bulrush, cattail’ and contemporary Japanese *kamasu* ‘straw bag’ (Robbeets, 2005, p. 201), as well as Tarama-Miyako *kamasɨ* and *kamasu* in a couple of modern Kyushu varieties. The derivation of **kamage* is an innovation restricted to Ryukyuan and Kyushu. The word PKR **moi-zo:ke* ‘winnowing basket for separating chaff from grain’ is an innovation, compounded of PKR **moi* from PJ **məi* ‘winnowing basket’, reflected in OJ *mi_2_* ‘winnowing basket’ and an element **so:ke* from PJ **sawoke* ‘bamboo basket’, which has Mainland Japanese cognates in Kōchi, *soːke*, and in Gumma, *ɕoːgi* (Hirayama, 1992-93, pp. 1139–1144).

Other shared innovations are found in the vocabulary for animal consumption and fishing. The semantics of PJ **sisi* ‘animal for consumption, meat’, reflected in OJ *sisi* ‘animal; flesh, meat’ and *wi-no-sisi* ‘wild boar’ have become specialised to indicate ‘even-toed ungulates such as boar and deer’ in Ryukyuan as well in a number of Kyushu topolects (SI 3: row 79): Kagoshima *ɕiɕi* ‘boar’, Ōita *ɕiɕi* ‘boar’ and Hirara/ Nagama *sï:sï* ‘boar’. In his 16-17th-century dictionary of Kyushu Japanese, João Rodriguez recorded *ɕiɕi* with the meaning ‘boar or deer’. Similarly, in the Aira area of the Kagoshima prefecture, the item *ɕiɕɕi* indicates more generally an ‘even-toed ungulate’.

Ryukyuan and Kyushu topolects seem to have most in common in the area of fishing vocabulary. Ryukyuan apparently shares a phonological innovation with regard to PJ **iwo* ‘fish’, which developed into PKR **ijo*, reflected in most Ryukyuan languages in addition to Kami-Koshiki and a couple of topolects in the Kagoshima prefecture areas of Satsuma and Kimotsuki (SI 3: row 268). The common forms with a high front vowel in Kagoshima, Kami-Koshiki, Tanegashima and Ōita, underlying the reconstruction PKR **iriko* ‘fish scales’ (SI 3: row 283) suggest preservation of pJ **iroko* ‘fish scales’, reflected in OJ *iroko* ‘fish scales’, while contemporary Japanese *uroko* ‘fish scales’ represents a case of vowel assimilation.

As it is unclear whether PJ **mina* reflected in OJ *mina* > *nina* meaning ‘snail’ should be reconstructed with the meaning of ‘snail’ or ‘shellfish’, the common form PKR **mina* ‘shellfish’ (SI 3: row 696) may reflect either an innovation or a preservation.

Ryukyuan and Kyushu topolects further share fish names, such as PKR **amamu* ‘hermit crab’ (SI 3: row 1000), **(kata-)pira-iyo* ‘olive flounder’ (SI 3: row 287) and *ira ‘jellyfish’ (SI 3: row 1002), which are not attested in Mainland Japanese. Other maritime cognates that may go back to a common Kyushu-Ryukyuan ancestor include Miyako-Ryukyuan *guː* ~ *guru* ‘shellfish’ and Kodakara island *goroa* ‘sea cucumber’ (SI 3: row 204), Miyako-Ryukyuan *sɨgal* ~ *sɨgaz* or Nakijin-Okinawan *ɕigai* ‘webfoot octopus’ (*Octopus* *ocellatus*) and Tanegashima *sugaru* ‘long-armed octopus’ (*Octopus* *minor*) (SI 3: row 1008), Proto-Ryukyuan *ɕibi ‘cowry’ and Tanegashima and Satsuma peninsula *ɕibi* used to indicate a range of tuna subspecies (SI 3: row 997); ‘wrasse’, PKR *kusabi (SI 3: row 1010), ‘sunken reef rocks’, PKR *pise (SI 3: row 1007), and PKR *abo (SI 3: row 996), whose reflex *abo* means ‘cliff’ in modern Tanegashima and Naka-Koshiki, while Miyako-Ryukyuan *abu* indicates ‘cave’, ‘crater’, ‘hole in the ground’, or ‘crack in the reef’.

Concerning ‘sunken reef rocks’, Uemura (1997, p. 315) reports that the word *piɕi* ‘an offshore sandbank’ (必至) is attested as a word of the Hayato tribe in the 8th century gazetteer of Ōsumi province (the *Ōsumi fudoki*) and analyses the word as having a likely Japonic etymology. Apparently, this claim of Japonic origins of the Hayato word was first published in a 1961 text by Kōji Uemura, cited by Karimata (2019, pp. 5–6); that same text also lists cognates of *piɕi* such as Kimotsuki (Ōsumi Peninsula) *hiɕi* and Tokara *fuse* ‘sunken reef rocks’. It is also worth mentioning that the Ainu language has the word *piɕ* ‘beach’. While jumping to conclusions based on just this one comparandum would be premature, this correspondence with the ‘Hayato language’ and Proto-Ryukyuan *pise* appears to be an indication that the ‘Hayato’ may have spoken a language related to Ainu, and consequently, that PKR has a Jōmon substratum.

Shared Kyushu-Ryukyuan vocabulary can also be observed in names for wild plants, such as ‘Asian taro’ (SI 3: row 994) and ‘wild grape’ (SI 3: row 1009).

By contrast, Ryukyuan names for trade goods from before the 12th century, such as ‘sulphur’ (SI 3: row 1006), ‘turban shell’ and ‘*Bischofia* *javanica’* (SI 3: row 995) are all of Japonic origin. ‘Sulphur’ and ‘*Bischofia* *javanica’* may have respectively descended from Proto-Japonic **yuwapu* and **akapoki*, while ‘turban shell’ is an obvious loan from Sino-Japanese *yakōgai*, and one scarcely attested in modern Ryukyuan (Shika-Ishigaki *jafungai* being a rare example). This is a strong indication that in trade situations the names assigned to Ryukyuan goods were Japonic and did not belong to an indigenous (Jōmon) language.

In sum, the agricultural vocabulary of Proto-Kyushu-Ryukyuan is largely continuous with that of its Proto-Japonic predecessor, even if there are a few common innovations shared by Kyushu and Ryukyuan topolects only. This observation confirms that the Proto-Kyushu-Ryukyuan speakers were farmers who inherited agriculture from their Japonic ancestors. Compared to other areas of subsistence, it is clearly seafood-related vocabulary that reveals the most copiously shared traits between Kyushu and Ryukyuan as opposed to other Japonic topolects. This is the case in spite of the fact that Kyushu and the Ryukyus belong to different climate zones with accordingly different marine fauna, which might invite a distinct labelling of such animals in the respective areas. The proportion of innovations in the shared Kyushu-Ryukyuan vocabulary in this semantic domain permits the conclusion that the Kyushu-Ryukyuan farmers developed a more maritime, seafaring culture after the break-up of Proto-Japonic.

***2. Shared innovations in phonology***

Turning to phonology, we can find a number of features shared between Kyushu topolects and Ryukyuan that are missing in other Mainland Japonic topolects.

Some Kyushu topolects and Ryukyuan languages preserve the distinction between PJ **ui* and **ǝi*, which merged into OJ *i_2_* and *i* in contemporary Mainland Japanese (Igarashi, 2020; e.g. SI 3: rows 901, 960) OJ *ki_2_* ‘tree’, *mi_2_* ‘winnowing basket’, PR **ke:* ‘tree’ vs. **moi* ‘winnowing basket’ and Kyushu (Kumamoto, Ōita, Fukuoka) *mi:*). However, since this is a case of retention rather than innovation, it is not the most convincing support for the proposed clustering between Kyushu and Ryukyuan.

Nevertheless, we also find the following shared innovations. First, whereas voiced obstruents go back to nasal obstruent clusters in Proto-Japonic (Frellesvig & Whitman, 2008) and can only rarely be reconstructed word-initially, we see an increased frequency of word-initial voiced obstruents in Kyushu and Ryukyuan. Usually, such words do not have obvious Japonic cognates. Examples include Kagoshima/Morokata *binta* ‘head’, Kagoshima *bebu* ‘bull’, *biki* ~ *bikkjon* ~ *bizzu* ‘frog’, Yakushima *baigaɕɕa*, Tokara *ibaɕi* and Tanegashima *baɕi-gaɕira* ‘Asian taro’ (*Alocasia odora/ indica*), along with cognates in Miyako *bivgassa*, *bivvulgassa*, *blblgassa* and Ishigaki/ Aragusuku *bjuːrɨ* of the same meaning, Tanegashima *gadʑabu* ‘a gnat’ cognate with Miyako *gadzam* ‘mosquito’, and Morokata *goro* ‘louse’ and its Kodakara cognate *goroa* ‘sea cucumber’.

The lack of initial voiced obstruents in Japanese and hence initial nasal clusters in Proto-Japonic is a prototypical characteristic that mirrors the typological feature of other Transeurasian languages not to allow for consonant clusters in initial position (Robbeets, 2017). Therefore, the increased frequency of word-initial voiced obstruents in Kyushu and Ryukyuan can be viewed as a shared innovation.

The item ‘fish’ (SI 3: row 268), PJ *iwo, underwent a PKR innovation to *ijo, which is reflected in Ryukyuan, Kami-Koshiki and Satsuma Peninsula cognates of this lexeme. A shared phonological innovation is also very likely observed in the items ‘snake’ (PKR *pajbu, SI 3: row 734) and ‘spider’ (PKR *kobu, SI 3: row 773). Shared innovative reflexes of the second syllable are observed in Ryukyuan, Kami-Koshiki and Tokara islands for ‘snake’ and broadly in Kyushu for ‘spider’. They reflect the vowel *u as opposed to reflexes of *i or *e in ‘snake’ (modern Japanese *hebi*) and *o in ‘spider’ (modern Japanese *kumo*).

Other shared innovations have been proposed by de Boer (2020, pp. 49-53), including the automatic lengthening of vowels in monosyllables, the merger of the tone classes 2.2. with 2.1 and 3.2 with 3.1 shared by the Kagoshima dialect and Ryukyuan, the shift from mora-based tone to word-tone in west and south Kyushu and Ryukyuan, and the split in the tone classes 2.4/5 and 2.3 in Ryukyuan and the Kishima dialect of Kyushu.

***3. Shared innovations in morphosyntax***

In the nominal morphology, some Kyushu topolects and Ryukyuan languages share common forms, such as PKR *-*ba* for the accusative case marker and PKR *-*kapje* ~ **ni kapje* for the allative/locative case (see Igarashi, 2017, p. 12 for the latter).

It has been suggested that the Mainland Japanese accusative marker -*o* and the topic particle *wa* < PJ **pa* are divergent developments of the same etymon and that -*o* is an assimilation of -*wa* < PJ *-*pa*, which is preserved as *-*ba* in Kyushu, Ryukyuan and some Tōhoku dialects and is cognate with the Tungusic accusative -*wa* ~ -*wǝ* (Itabashi, 1988; Martin, 1988). The Old Japanese emphatic accusative -*womba* also involves the accusative -*wo* and topic -*pa*. From this perspective, the common reflex in Kyushu and Ryukyuan can be viewed as a common retention.

In the light of the directive case marker OJ -*pe_1_*, which is the result of grammaticalisation of the noun *pe_1_* < PJ **pje*  ‘side’, the directive PKR *-*kapje* appears to be an innovative compound consisting of an older locative **ka*, grammaticalised from a noun meaning ‘place, location’ and **pje* ‘side’. Its extended form *-*nikapye* prefixes the dative marker *-*ni*. The modern reflexes of *-*nikapje* ~ *-*kapyje* include Shuri-Okinawan -*nkeː*, Miyako -*nkai*, Kagoshima and Miyazaki -*ke*, Koshiki -*kjaː*, Kumamoto and Nagasaki -*gjaː*, Fukuoka -*geː* (Hirayama, 1992–1993; Jarosz, 2021, p. 61).

From a structural perspective, Kyushu and Ryukyuan tend to preserve the original Japonic distinction between interrogative markers used in yes-no questions and those used in wh-questions, reflected in the use of Old Japanese -*ja* for the former case and -*ka* for the latter. However, some formally specific question markers, such as the use of the interrogative marker *-*na* in yes-no questions, may represent common innovations shared between Ryukyuan languages and some Kyushu topolects in the Kagoshima area and its outlier islands such as Kami-Koshiki.

In addition, we find a number of shared grammaticalisations limited to Kyushu topolects and Ryukyuan languages. In Old Japanese, the noun *kara* ‘clan, relationship, character’ had just started to grammaticalise into an ablative case marker -*kara*, but Kyushu and Ryukyuan both grammaticalised the noun into a motion instrumental marker. The marker is used to indicate the means of motion (mostly vehicles) and combines this function with the ablative. It is found among others in Kagoshima (-*kaa*), Miyako (-*kara*; Hirayama, 1993-4, pp. 3342–44), and in the Chikugo area (-*kara*; Matsuda, 1991, p. 208).

Kyushu and Ryukyuan also share the grammaticalisation of the verbs PJ **nar*- ‘to become’, reflected in OJ *nar*- ‘to become’ and PJ **ǝyǝ-s*- ~ *ǝpǝ-s*- ‘to grow, become old’, reflected in OJ *oyo_2_si* ‘becoming old’, MJ *oyos*- ‘get old’ and MJ *opos-* ‘to complete, to succeed at’ into the ability potential auxiliaries PKR **nar*- and **oyos*-. They further share a purposive construction consisting of a converb followed by the directive marker *-*ka* or *-*kapye* and a motion verb.

**References**

de Boer, E. (2020). The classification of the Japonic langauges. In M. Robbeets & A. Savelyev (Eds.), *The Oxford guide to the Transeurasian languages* (pp. 40-58). Oxford University Press.

Frellesvig, B. & Whitman, J. (2008). Evidence for seven vowels in proto-Japanese. In B. Frellesvig & J. Whitman (Eds.) *Proto-Japanese: Issues and prospects* (pp. 15–41) Amsterdam: Benjamins.

Hirayama T. (Ed.) (1992–1993). *Gendai nihongo hōgen daijiten*, vols. 1–6. Tokyo: Meiji Shoin.

Igarashi, Y. (2017). *Kyūshū: Ryūkyū dōgengo chōsa-hyō* [A questionnaire of Kyūshū- Ryūkyū cognates], Paper presented at the Hitotsubashi graduate school “all day seminar”, 12 September 2017.

Igarashi Y. (2018). Bunkigakuteki hōhō-ni motozuita nihongo/ryūkyūgo shohōgen-no keitō bunrui-no kokoromi. [Paper presented at a NINJAL symposium *Fiirudo-to bunken-kara miru nichiryūshogo-no keitō-to rekishi* in Tokyo, 23 December 2018.]

Itabashi, Y. (1988). A comparative study of the Old Japanese accusative case suffix *wo* with the Altaic accusative case suffixes. *Central Asiatic Journal*, *32*, 193-231.

Jarosz, A. (2019). Non-core vocabulary cognates in Ryukyuan and Kyushu. In H. Aoi (ed.), *Proceedings of International Symposium ‘Approaches to Endangered Languages in Japan and Northeast Asia (Poster Session)*’ (pp. 8–29). Tokyo: Research Institute for Languages and Cultures of Asia and Africa, Tokyo University of Foreign Studies.

Jarosz, A. (2021). Old Japanese post-nasal non-back close vowels in a comparative perspective. *International Journal of Eurasian Linguistics*, *3*, 44–76.

Karimata S. (2019). Gengo-kara kangaeru Kyūshū-kara Ryūkyū-e-no hito-no idō. Gengo-to bumpō-kara idō-no jiki-o kangaeru / Investigating migrations from Kyushu into the Ryukyus through language. *Kokusai Ryūkyū Okinawa Ronshū* / *International Review of Ryukyuan and*

*Okinawan Studies*, 8, 1–10.

Katō, M. (1977). Hōgen kukakuron. In S. Ōno & T. Shibata (Eds.), *Iwanami kōza Nihongo 11: Hōgen* (pp. 41–82). Tokyo: Iwanami shoten.

Lee, S., & Hasegawa, T. (2011). Bayesian phylogenetic analysis supports an agricultural origin of Japonic languages. *Proceedings of the Royal Society B*, *278*, 3662-3669.

Martin, S. E. (1988). *A Reference Grammar of Japanese*. Tokyo: Tuttle.

Matsuda Y. (1991). *Chikugo hōgen jiten*. Kurume: Kurume Kyōdo Kenkyūkai.

Nohara, M. (1979–1983). Ryūkyū hōgen-to Kyūshū hōgen-to-no hikaku. Parts I-V. *Okinawa Kokusai Daigaku Bungakubu Kiyō, Kokubungaku-hen*, 8, 1-16; 9, 1-20; 10, 1-16; 11, 1-16; 12, A1-A14.

Pellard, T. (2015). The linguistic archaeology of the Ryukyu Islands. In P. Heinrich, S. Miyara & M. Shimoji (Eds.), *Handbook of the Ryukyu languages: History, structure and use* (pp. 13-37). De Gruyter.

Robbeets, M. (2005). *Is Japanese related to Korean, Tungusic, Mongolic and Turkic?* Wiesbaden: Harrassowitz.

Robbeets, M. (2017). Farming language dispersal hypothesis: food for thought. In M. Robbeets & A. Savelyev (Eds.), *Language dispersal beyond farming* (pp. 1–24). Amsterdam: Benjamins.

Robbeets, M. et al. (2021). Triangulation supports agricultural spread of the Transeurasian languages. *Nature*, 599, 616-621.

Uemura, Y. (1997). Ryūkyū rettō-no gengo, sōsetsu. In Kamei, T., Kawano, R., & Chino, E., eds., *Gengogaku daihyakka serekushon. Nihon rettō-no gengo* (pp. 311–353). Tokyo: Sanseidō.

Unger, J. M. (2009). *The role of contact in the origins of the Japanese and Korean languages*. University of Hawai‘i Press.

1. Apart from these, Nohara (1979–83) is an extensive list of shared Kyushu and Ryukyuan vocabulary. This is, however, simply a set of lexical lookalikes without any diachronic analysis, not aiming to differentiate between loanwords and cognates, and as a consequence, a bulk of it is formed by early modern Kyushu loanwords into Okinawan. [↑](#footnote-ref-1)
